# Illustrating and homology modeling the proteins of the Zika virus

**DOI:** 10.12688/f1000research.8213.2

**Published:** 2016-09-01

**Authors:** Sean Ekins, John Liebler, Bruno J. Neves, Warren G. Lewis, Megan Coffee, Rachelle Bienstock, Christopher Southan, Carolina H. Andrade

**Affiliations:** 1 Collaborations in Chemistry, Fuquay-Varina, NC, USA; 2 Collaborations Pharmaceuticals Inc., Fuquay-Varina, NC, USA; 3 Collaborative Drug Discovery Inc, Burlingame, CA, USA; 4 Art of the Cell, Guilford, CT, USA; 5 LabMol - Laboratory for Molecular Modeling and Drug Design, Faculty of Pharmacy, Federal University of Goias, GO, Brazil; 6 Department of Medicine, Washington University School of Medicine, St Louis, MO, USA; 7 The International Rescue Committee, New York, NY, USA; 8 RJB Computational Modeling LLC, Chapel Hill, NC, USA; 9 Centre for Integrative Physiology, University of Edinburgh, Edinburgh, UK

**Keywords:** Aedes mosquito, dengue virus, drug discovery, ebola virus, flavivirus, microcephaly, yellow fever, Zika virus

## Abstract

The Zika virus (ZIKV) is a flavivirus of the family *Flaviviridae*, which is similar to dengue virus, yellow fever and West Nile virus. Recent outbreaks in South America, Latin America, the Caribbean and in particular Brazil have led to concern for the spread of the disease and potential to cause Guillain-Barré syndrome and microcephaly. Although ZIKV has been known of for over 60 years there is very little in the way of knowledge of the virus with few publications and no crystal structures. No antivirals have been tested against it either *in vitro* or *in vivo*. ZIKV therefore epitomizes a neglected disease. Several suggested steps have been proposed which could be taken to initiate ZIKV antiviral drug discovery using both high throughput screens as well as structure-based design based on homology models for the key proteins. We now describe preliminary homology models created for NS5, FtsJ, NS4B, NS4A, HELICc, DEXDc, peptidase S7, NS2B, NS2A, NS1, E stem, glycoprotein M, propeptide, capsid and glycoprotein E using SWISS-MODEL. Eleven out of 15 models pass our model quality criteria for their further use. While a ZIKV glycoprotein E homology model was initially described in the immature conformation as a trimer, we now describe the mature dimer conformer which allowed the construction of an illustration of the complete virion. By comparing illustrations of ZIKV based on this new homology model and the dengue virus crystal structure we propose potential differences that could be exploited for antiviral and vaccine design. The prediction of sites for glycosylation on this protein may also be useful in this regard. While we await a cryo-EM structure of ZIKV and eventual crystal structures of the individual proteins, these homology models provide the community with a starting point for structure-based design of drugs and vaccines as well as a for computational virtual screening.

## Introduction

All flaviviruses are spherical and contain a genome of approximately 11kb that functions as mRNA and encodes a polyprotein that leads to 10 proteins ^1^. Examples include dengue virus, yellow fever and West Nile virus ^2^. The recent pandemic of ZIKV occurring in South America, Latin America, the Caribbean and in particular Brazil spread by the *Aedes* mosquito has awakened dormant interest in this flavivirus which is a mild dengue-like disease ^3^. However several documented cases of Guillain-Barré syndrome and other neurologic conditions represent important complications of the disease. In recent weeks the extent of the disease has also become apparent as new discoveries and announcements are made almost daily. Though clearly we have a considerable number of significant gaps in our knowledge which need addressing ^4^.

The most concerning issue however is microcephaly observed in women who had ZIKV during pregnancy. There have been multiple cases of ZIKV found in fetal or newborn brain tissue that had signs of prenatal damage. The virus seems to have neurotropism in fetal brains, which may account for the presumed association between the infection and microcephaly ^5, 6^. The fetus in the recent case study had microcephaly with calcifications and ZIKV was found in the brain ^6^. The ZIKV strain was identified as from French Polynesia (GenBank accession number KJ776791) and several polymorphisms were noted in the NS1, NS4B and FtsJ like methyltransferase regions. While the findings are not absolute proof that ZIKV causes microcephaly, the evidence from this case report strengthens the linkage ^7^. Experts involved in the decision on the World Health Organization determined Public Health Emergency of International Concern (PHEIC) recommended the need for more research into the microcephaly link and need for an animal model to be developed. This group also interestingly called for open data sharing ^8^. Early work 45 years ago in inoculated newborn mice showed that ZIKV had neurological effects, enlarging astroglial cells and destroying pyriform cells. At the same time virus formation within the endoplasmic reticulum was also visualized ^9^. We are not aware of any studies of effects of ZIKV on human brain or brain cells. Localization of such viruses to the brain is not unusual for flaviviruses i.e. West Nile virus and this tropism may arise from viral binding to glycosaminoglycans, as has been observed for dengue virus in human microvascular endothelial cells ^10^. Heparan sulfate and the C-type lectin DC-SIGN (dendritic cell-specific intercellular adhesion molecule 3-grabbing nonintegrin) are well characterized attachment structures for flaviviruses on cells. Interfering with glycan binding is one potential approach to preventing virus entry. Another is to acidify the endosome as has been demonstrated *in vitro* with chloroquine for dengue virus infection ^2^. Several entry and adhesion factors, including DC-SIGN, Tyro3, and AXL as well as others, have been shown to permit ZIKV entry in human skin cells ^11^.

The routes for transmission of ZIKV besides mosquito are of some concern. Recent US CDC guidance to pregnant women describes precautions against sexual transmission of ZIKV ^12^ and that the virus can persist for up to 12 weeks ^13^. Possible ZIKV transmission through blood transfusion in French Polynesia was described by detecting the virus in 3% of asymptomatic blood donors ^14^. Given how widespread ZIKV has become, there is a risk of depleting the blood supply, if donation after potential virus exposure is deferred. Methods have also been developed to inactivate ZIKV in plasma using amotosalen and UVA illumination ^15^. There are issues with detection of ZIKV as a false positive dengue NS1 antigen test in a traveler to Switzerland was found to have the virus later. Therefore, cross-reactivity appears to be an issue in detection ^16^ and this also suggests the need for better diagnostics to be developed.

Structural knowledge of the ZIKV proteins may allow us to understand exposed epitopes which will facilitate the development of specific diagnostic reagents that differentiate it from dengue and other flaviviruses. Furthermore, open sharing of the three-dimensional arrangement of viral surface proteins could allow the mapping of potential neutralizing epitopes, guiding efforts to rationally design effective vaccines. We recently developed a preliminary model for ZIKV glycoprotein E based on the dengue virus glycoprotein E late stage fusion intermediate as a trimer ^4^. We now provide homology models of the glycoprotein E based on a dimer structure as well as attempts at modeling the other proteins in ZIKV. We have also investigated the likely glycosylation sites of the ZIKV envelope glycoprotein. Glycosylation may obstruct the binding of antibodies, or block access to potential underlying peptide antigens, so glycans may be an important consideration in diagnostic and vaccine development. Previously, glycosylation analysis using several computational tools predicted mammalian *N*-linked glycosylation at Asn-154 in most ZIKV strains ^17^, a site known to be important in other flaviviruses. *N*-glycosylation of the dengue envelope glycoprotein at two sites has been shown to mediate interactions with DC-SIGN ^18^. Insect cell expression of dengue shows both high-mannose and complex glycans ^19^. However, flavivirus glycosylation in model systems such as insect cell culture and mammalian tumor cell lines may not represent the true infective insect and mammalian glycoprotein. Mammalian *O*-linked glycosylation was predicted at Thr-245 and Thr-381 in some isolates ^17^. Yet, reliable prediction is hampered by a lack of *O*-glycosylation consensus sequences and preferences for *O*-glycosylation driven by structural characteristics ^20^.

Understanding the three-dimensional structure of antigenic ZIKV proteins may help accelerate the development of antibodies for diagnostics and rationally designed vaccines. In addition, the comparison of the assembled surface glycoprotein of ZIKV with that of dengue virus may help understand the accessible epitopes for the development of anti-flaviral vaccines in general. There is considerable prior work including structure-based design and virtual screening for dengue, yellow fever and other related flaviviruses to develop antivirals to target envelope glycoproteins ^21– 26^, guanylyltransferase ^27^, capsid protein NS3 helicase, NS2B-NS3 protease and NS5 polymerase ^28, 29^, as well as whole cell screens ^30^ which have produced many molecules potentially useful against ZIKV *in vitro*. Early work has only tested a small number of FDA-approved drugs against ZIKV including (EC _50_ in parenthesis) interferon (34.3 IU/ml), ribavirin (143 ug/ml), 6-azauridine (1.5 ug/ml) and glycyrrhizin (384 ug/ml) ^31^. A recent paper by Hamel *et al*., from 2015 also showed interferon inhibited ZIKV replication in primary skin fibroblasts ^11^.

However, the use of compounds against ZIKV should take into account the treatment of pregnant women, and many of the potential options are unsuitable for use in pregnancy because of toxicity and/or teratogenicity. Despite limited human data, the available data in animal models suggests caution. Azauridine is highly toxic to the fetus in model systems (for example 32). Ribavirin is not recommended for use in pregnancy due to embryotoxic and teratogenic effects ^33^. Interferon is a potential abortifacient ^34^. We also need to consider the treatment of fetus as well as children (that might become infected after birth) and the relatively small subsection of FDA approved drugs that are approved for pediatric use ^35^. Therefore, alternative potential drugs for ZIKV are needed. The risks of medication use in pregnancy are notable. In particular, these include teratogenicity concerns. There is also the issue that the disease does not usually pose a direct risk to pregnant women themselves, so it’s important any drug, which will not be improving their own health, does not damage their health. Pregnancy can also create increased risks and liver problems, a particular concern with any new drug as it can also affect drug distribution. In pregnancy, a ZIKV infection at 13 weeks gestation was coupled with persistent virus in a fetus at 32 weeks ^6^. Treatment of symptomatic pregnant women may reduce risks of transmission to the fetus. A potential drug for ZIKV could also protect fetuses from damage by reducing transmission in the general population. If the drug appeared to reduce the duration of symptoms (which though mild can be annoying) and in turn reduced viral load, reducing the chance of transmission, this could benefit them. For example, cholera patients are often given antibiotics to reduce transmission. Also those with the flu are prescribed Tamiflu/Oseltamivir to reduce the duration of symptoms, minimize the severity of symptoms, but its use might also reduce transmission of flu in the general population. Since ZIKV has been found in semen many weeks after symptoms resolve ^12^, treatment of male partners may reduce viral load and reduce the long term risk of transmission.

To help accelerate drug discovery through computational analysis, we have now developed homology models of ZIKV proteins that may serve as potential drug and vaccine targets. To complement high-throughput screening efforts, we could perform virtual screening against the proteins in ZIKV. While there are crystal structures for proteins from dengue ^36, 37^, yellow fever, West Nile virus and other flaviviruses ^38– 44^ there are (to date) none for ZIKV. Therefore we are limited to generating homology models, although the close evolutionary relationships between flavivirus and their component proteins and genomes represents a valid approach ^45^.

## Methods

### Protein sequence alignment

As a prelude to modeling we assessed what 3D structural data had significant identity scores to a representative ZIKV polyprotein. For this we chose UniProtKB Q32ZE1_9FLAV. While we have requested the promotion of this entry to the Swiss-Prot expert review level it has been selected as the representative sequence for the UniRef90_Q32ZE1 entry that currently clusters 108 ZIKV individual sequence entries at 90% (or above) amino acid identity. We then performed a BLAST search of this against Protein Data Bank (PDB) sequence entries. Protein BLAST analysis was also performed for each ZIKV protein sequence ^46^ to identify the closest proteins ^47^ and understand potential evolution.

### Homology modeling

The amino acid sequences of ZIKV strain (GenBank accession number KJ776791 ^48^) were retrieved from the GenBank database ^49^ and used as targets for homology modelling using the SWISS-MODEL server ^50, 51^. The latter performed the target-template sequence alignment after searching the putative X-ray template proteins in PDB for generating the 3D models for all target sequences. The best homology models were selected according to Global Model Quality Estimation (GMQE) and QMEAN statistical parameters. GMQE is a quality estimation which combines properties from the target-template alignment. The quality estimate ranges between 0 and 1 with higher values for better models. QMEAN4 scoring function consisting of a linear combination of four structural descriptors as described elsewhere in more detail ^52, 53^. The pseudo energies returned from the four descriptors are related to what we would expect from high resolution X-ray structures of similar size using a Z-score scheme. Further, built models were exported to the SAVES server Version 4 ^54^ and their overall stereochemical quality, including backbone torsional angles through the Ramachandran plot, was checked according to PROCHECK ^55^. Lastly, each model was submitted to an energy minimization protocol, using the Smart Minimizer algorithm in Discovery Studio version 4.1 (Biovia, San Diego, CA).

### Site of glycosylation prediction

Mammalian *N*-glycosylation sites were predicted for glycoprotein E by submitting the sequence to web-based tools namely N-GlycoSite ^56^, GlycoEP ^57, 58^ and NetNGlyc Version 1.0 ^59^.

### Illustration for Zika virion and animation

The Zika virion illustrations were created by combining the homology model of the envelope ZIKV glycoprotein E with the symmetry data from the dengue virus envelope. PDB ID:1K4R ^60^ contains the coordinates for three copies of the protein subunit of the dengue virus envelope, along with the symmetry data necessary to create the 180-subunit icosahedral structure of the complete viral envelope. The PyMOL Molecular Graphics System, Version 1.7.6.0. Schrödinger, LLC. was used to export the surface models of the three proteins in .obj format. Then they were imported into Lightwave 3D (NewTek, San Antonio, TX) where the symmetry data was used to instance copies of the model into the icosahedral envelope. The entire structure was copied several times and lighting applied as a surfacing effect to create a visually pleasing composition, and the image rendered out.

The next step was to import into Pymol the homology model of the ZIKV envelope protein which was homology modeled using PDB ID: 3P54 (from Japanese Encephalitis Virus) as a template. A surface model of this protein was exported from Pymol as an .obj, and imported into Lightwave in place of the dengue model, using the same symmetry operators to create the envelope array. Everything else about the picture was left the same (color, composition, lighting, etc...) to allow the structural differences to be more apparent, and that image rendered out as well.

The last step was to overlay the detailed area of the two images and create an animated gif to flip back and forth between the two images of ZIKV and dengue, again to allow the differences to be more clearly seen. The structure of the Zika virion could be explored in a similar manner, using known data from other flaviviruses as a guide.

### ZIKV glycoprotein E homology model conformation comparison

The immature ^4^ and mature (this study) homology models for glycoprotein E were compared using the ‘align and superimpose’ proteins protocol in Discovery Studio Version 4.1 (Biovia, San Diego, CA).

## Results

### Sequence alignment across flaviviruses

A BLAST search of ZIKV polyprotein against PDB sequence entries shows the highest scoring matches with 55–70% sequence identity ( Supplementary material S1). Protein BLAST analysis of the individual ZIKV protein sequences show that many of the proteins are similar to the same protein from Spondweni virus in 12 out of 15 cases ( Table 1). Exceptions were: FtsJ which was closer to the Murray Valley encephalitis virus, NS1 which was more similar to dengue virus 3, and glycoprotein E which was closest to the dengue virus 1 protein. These results are in general accordance with whole sequence analyses ^45^.

**Table 1.** Protein BLAST search results - closest non-ZIKV proteins.

Protein	FASTA	Closest sequence	Coverage	E value	Identity (%)
NS5	AHZ13508.1 (2,772..3,412)	RNA-dependent RNA polymerase NS5 [Spondweni virus]	100	0	77
FtsJ	AHZ13508.1 (2,575..2,746)	Chain A, Crystal Structure Of The Murray Valley Encephalitis Virus Ns5 2'-O Methyltransferase Domain In Complex With Sah (Monoclinic Form 1)	99	1e91	76
NS4A	AHZ13508.1 (2,124..2,267)	polyprotein [Spondweni virus]	100	3e68	75
HELICc	AHZ13508.1 (1,859..1,975)	nonstructural protein NS3 [Spondweni virus]	100	5e61	86
Peptidase S	AHZ13508.1 (1,520..1,670)	nonstructural protein NS3 [Spondweni virus]	99	2e81	82
DEXDc	AHZ13508.1 (1,683..1,828)	nonstructural protein NS3 [Spondweni virus]	99	2e79	83
NS2B	AHZ13508.1 (1,376..1,502)	nonstructural protein NS2B [Spondweni virus]	100	1e56	76
NS2A	AHZ13508.1 (1,158..1,372)	nonstructural protein NS2A [Spondweni virus]	100	1e64	55
NS1	AHZ13508.1 (796..1,148)	polyprotein [Dengue virus 3]	100	2e136	56
E stem	AHZ13508.1 (698..794)	polyprotein [Spondweni virus]	100	1e38	67
Glycoprotein M	AHZ13508.1 (216..290)	membrane glycoprotein m [Spondweni virus]	100	1e31	67
Propeptide	AHZ13508.1 (126..214)	protein pr [Spondweni virus]	100	2e35	61
Capsid	AHZ13508.1 (6..122)	anchored capsid protein C [Spondweni virus]	96	4e41	68
Glycoprotein E	AHZ13508.1 (291..592) AHZ13508.1 (601..693)	envelope protein E [Dengue virus 1]	100	3e165	57
NS4B	AHZ13508.1 (2,270..2,514)	nonstructural protein NS4B [Spondweni virus]	100	2e130	82

### Homology modeling

**Figure 1.**
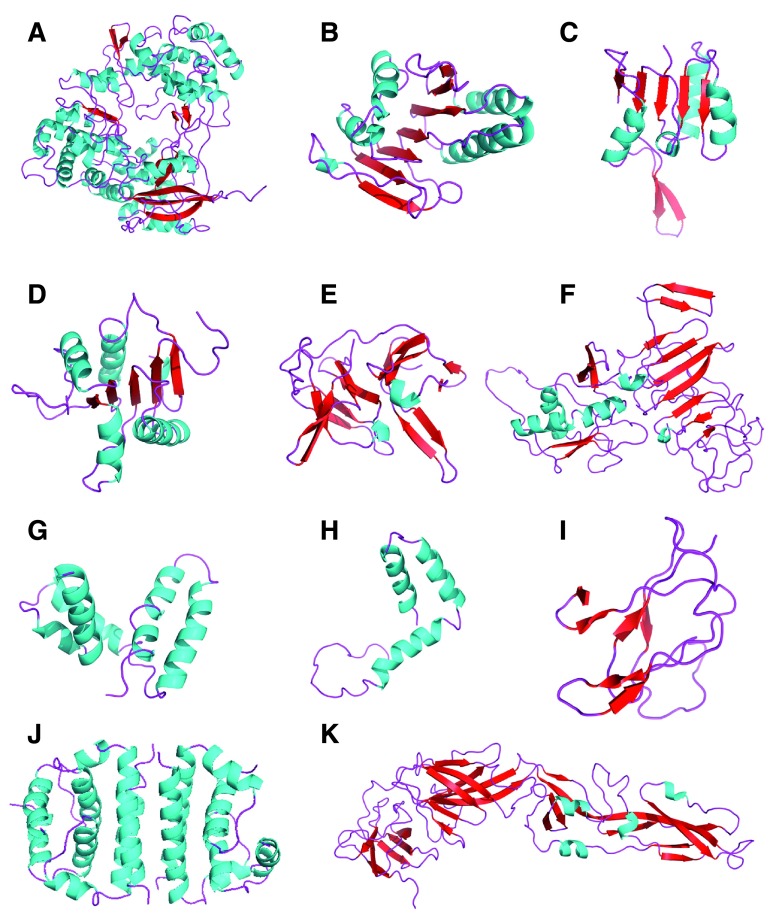
Selected ZIKV NS5 ( **A**), FtsJ ( **B**), HELICc ( **C**), DEXDc ( **D**), Peptidase S7 ( **E**), NS1 ( **F**), E Stem ( **G**), Glycoprotein M ( **H**), Propeptide ( **I**), Capsid ( **J**), and Glycoprotein E ( **K**) homology models (minimized proteins) that had good sequence coverage with template proteins developed with SWISS-MODEL.

**Figure 2.**
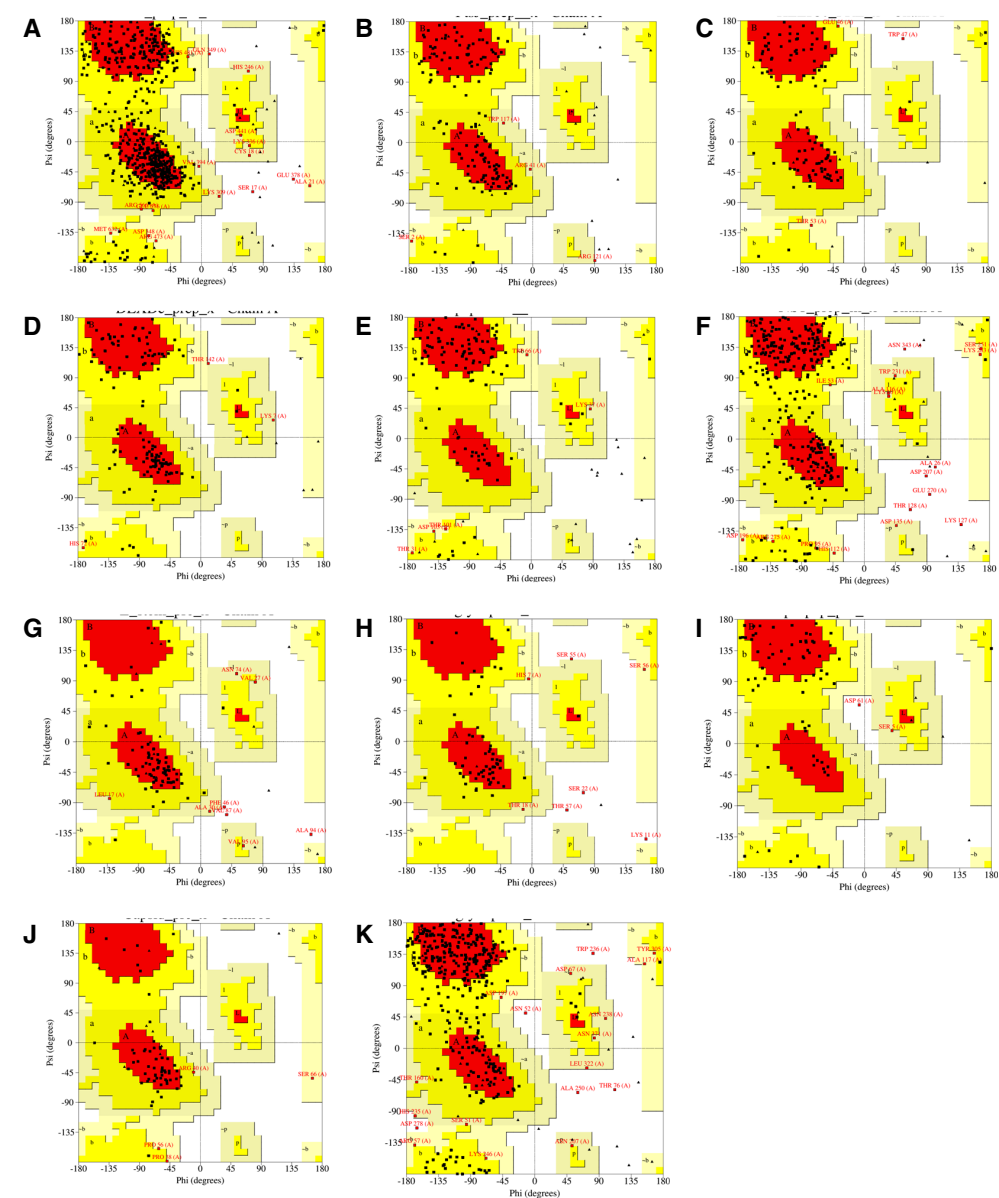
Ramachandran plots for ZIKV NS5 ( **A**), FtsJ ( **B**), HELICc ( **C**), DEXDc ( **D**), Peptidase S7 ( **E**), NS1 ( **F**), E Stem ( **G**), Glycoprotein M ( **H**), Propeptide ( **I**), Capsid ( **J**), and Glycoprotein E ( **K**) obtained by PROCHECK, showing the dihedral angles Psi and Phi of amino acid residues. Red represents most favored regions; yellow represents additional allowed regions; beige represents generously allowed regions; and white areas are disallowed regions.

The SWISS-MODEL server was used to generate alignments ( Supplementary material S2) and homology models for all Zika proteins ( Table 2, Figure 1, Supplementary material S3). First, we selected suitable template protein structures in PDB, observing the following criteria: the template should have a high coverage (i.e., > 65% of target aligned to template) and sequence identity >30%. Then, we used GMQE and QMEAN4 scoring function as an initial criteria to discriminate good from bad models. Acceptable alignment values and higher GMQE and QMEAN4 scores were obtained during modeling, suggesting statistically acceptable homology models were generated for 11 proteins: NS5, FtsJ, HELICc, DEXDc, peptidase S7, NS1, E stem, glycoprotein M, propeptide, capsid, and glycoprotein E ( Table 2, Figure 1 and Figure 2). The Ramachandran plots for these 11 proteins provide further evidence of their acceptability ( Figure 2). On the other hand, because of low GMQE scores and of low coverage observed in X-ray template proteins available in the PDB, homology models for NS4B, NS4A, NS2B, and NS2A proteins appeared to have limitations regarding active sites and epitopes and they could not be validated.

**Table 2.** Summary of ZIKV homology models and statistical validation. The global and per-residue model quality has been assessed using the QMEAN scoring function ^53^. For improved performance, weights of the individual QMEAN terms have been trained specifically for SWISS-MODEL ^50, 51, 71– 73^. GMQE = Global Model Quality Estimation, QMEAN4 is a scoring function consisting of a linear combination of four structural descriptors as described elsewhere in more detail ^52, 53^.

Zika Protein	Template			Coverage	Sequence Identity	GMQE	MEAN4	PROCHECK analysis			
	Organism	Protein	PDB code					Most favored regions	Additional allowed regions	Generously allowed regions	Disallowed regions
NS5	Japanese encephalitis virus	NS5 ^61^	4K6M	100%	53%	0.89	-2.80	66.3%	30.9%	2.3%	0.9%
FtsJ	West Nile virus	methyltransferase ^62^	2OY0	99%	54%	0.94	-1.23	69.5%	27.7%	2.1%	0.7%
HELICc	Dengue virus	helicase/nucleoside triphosphatase catalytic domain ^63^	2BHR	100%	55%	0.94	-1.60	69.3%	27.7%	2.0%	1.0%
DEXDc	Murray Valley encephalitis virus	RNA helicase ^64^	2V8O	100%	54%	0.93	-0.92	70.3%	27.3%	1.6%	0.8%
Peptidase S7	West Nile virus	Ns2B-Ns3 protease ^65^	2YOL	100%	53%	0.92	-0.25	67.5%	28.3%	4.2%	0.0%
NS1	West Nile virus	non-structural protein 1 ^66^	4O6D	99%	48%	0.77	-4.09	58.4%	36.3%	3.3%	2.0%
E Stem	Dengue virus	capsid protein ^67^	3J2P	100%	46%	0.74	-8.02	62.5%	27.5%	6.3%	3.8%
Glycoprotein M	Dengue virus	cryo-EM structure of virus ^67^	3J27	100%	40%	0.73	-6.90	60.6%	28.8%	4.5%	6.1%
Propeptide	Dengue virus	precursor membrane protein-envelope ^68^	3C5X	87%	47%	0.74	-0.70	53.7%	43.3%	1.5%	1.5%
Capsid	West Nile virus	core (C) protein ^69^	1SFK	65%	42%	0.50	-3.54	69.5%	27.1%	3.4%	0.0%
Glycoprotein E	Japanese encephalitis virus	envelope glycoprotein ^70^	3P54	99%	47%	0.81	-3.76	63.5%	31.3%	3.7%	1.4%
NS4A	─	─		20%	29%	0.07	-1.75	─	─	─	─
NS2B	─	─		37%	48%	0.21	-0.49	─	─	─	─
NS2A	─	─		16%	31%	0.04	-3.10	─	─	─	─
NS4B	─	─		14%	30%	0.03	-2.81	─	─	─	─

After building of homology models, we performed an additional validation in order to explore stereochemical quality of dihedral angles phi against psi of amino acid residues in modeled structures and identify sterically allowed regions for these angles using PROCHECK analysis. The results shown in Table 2 and Figure 2 reveal that 58.4─70.3% residues of the modeled proteins are within the most favored regions (red), 27.1─43.3% residues of modeled proteins are within the additional allowed regions (yellow), 1.5─6.3% residues of modeled proteins are within the generously allowed regions (beige), and only 0.0─6.1% residues of modeled proteins are within the disallowed regions (white). These results showed that the overall stereochemical properties of the generated models were highly reliable and the models could be useful to future molecular modeling studies.

### Site of glycosylation prediction

Several web-based tools were used for *N*-glycosylation site predictions as it provides a more thorough approach. N-GlycoSite ^56^ suggested N154 as a single N-glycosylation site matching the N-X-S/T/C consensus sequence. The same site was identified by GlycoEP using BPP settings (binary profile of patterns) ^57, 58^ giving a score of 0.65/1.00. NetNGlyc ^59^ also gave the same predicted site, with a jury agreement of 6/9.

### Zika virion compared to dengue virion

**Figure 3.**
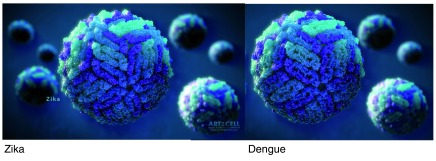
Comparison of Zika and dengue virion illustrations.

A qualitative analysis of the Zika virion (which was constructed based on the dengue virion) can be compared to the dengue cryo-EM virion ( Figure 3) and indicates that Zika appears to have slightly more raised ‘pimples’ on the surface. The glycoprotein E dimer in ZIKV also has a narrow ‘letter-box’ groove while the dengue virion has a bigger ‘pore‘ between the intersection of 5 dimers (5 fold axis). These differences are considerably more apparent in the animation ( Supplementary material S4). It is important to note that the differences may also be artefacts of the homology modeling approach and template used for modeling ZIKV glycoprotein E.

### ZIKV glycoprotein E homology model conformation comparison

**Figure 4.**
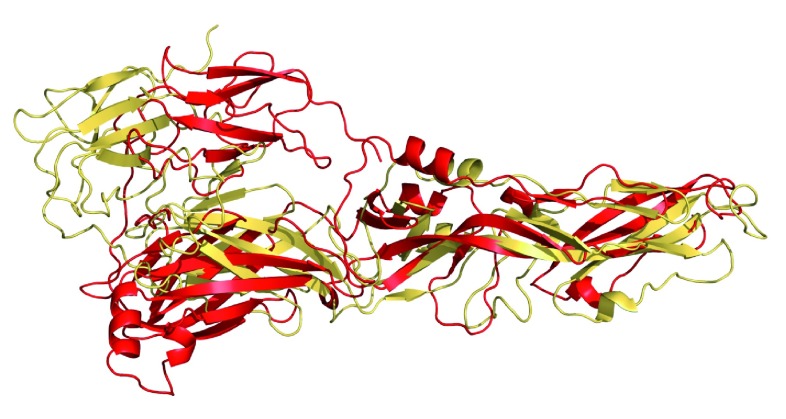
Overlap of ZIKV homology models for glycoprotein E, Yellow = mature conformation (this study) compared with the immature conformation (red) ^4^.

The homology models developed using two different templates namely the immature protein which was based on the dengue crystal structure 4gsx as a template ^50, 71– 73^ and the mature protein which was based on PDB ID:3P54 from Japanese encephalitis virus showed a large difference (RMSD 13.47Å) ( Figure 4). These proteins also demonstrate differences around the pocket used centered on the residues 270-277.

## Discussion

The genus Flavivirus consists of 70 viruses many of which can cause severe human disease. There have been few sequence analyses of ZIKV previously in comparison to other flaviviruses. The genus Flavivirus produces a monophyletic tree with ZIKV being closest to Spondweni virus ^74^ while mosquito borne, tick borne and no-vector viruses cluster separately ^45^. A BLAST analysis of all the ZIKV proteins in this study suggests for 12 of 15, their closest protein is in Spondweni virus ( Table 1). More often strain sequences are compared within ZIKV and these showed variations in the NS5 gene ^75^ and glycoprotein E ^17^. This is important as it would suggest perhaps targeting other proteins would have less issue with resistance or variability due to the strain of ZIKV.

If we are to address ZIKV in the short term while we await a vaccine we need to rapidly identify an antiviral, and preferably one that can be used against other related flaviviruses. Ideally we would need to treat pregnant women and provide them with prophylaxis that was safe to them and their fetus. Such an antiviral could also be used to reduce transmission in the population in general (by reducing viral load and symptoms and/or duration). As noted a decade ago and is still is true today, no antiviral drug is approved for any flavivirus to date ^76^. It has been suggested that one of the ways to target these viruses is to interfere with the NS2B/NS3 protease complex ^76^. Understanding of flavivirus proteins and other RNA viruses has benefited from the EU funded project VIZIER ^77^, in particular several West Nile virus, dengue virus and other flavivirus structures of NS3 or NS5 were solved during this project and allosteric inhibitor sites were identified on NS5 ^78^. Multiple pharmaceutical companies have worked on this target for HCV leading to clinical candidates like IDX320 ^79^, danoprevir (ITMN-191/R7227) ^80^, GS-9256 ^81^ and others ^82, 83^. The only HCV protease targeting FDA approved drug is simeprevir, TMC435 ^84, 85^ and its use is avoided in pregnancy. Other HCV protease compounds are in clinical trials or submitted for FDA approval including Ledipasvir (formerly GS-5885) ^86^. Testing these molecules against ZIKV *in vitro* would be useful.

We recently described 6 steps which could be taken to kick start research on ZIKV ^4^, one of which was to develop homology models for ZIKV proteins that are similar to those targeted by molecules that are also active against the dengue virus. Such an approach would then enable docking of compound libraries of known antivirals, FDA approved drugs or other compounds ^4^. Ideally generating homology models with a single tool may not be enough. In particular, for those proteins with low sequence identity the use of servers and methods that use threading may be worthwhile (e.g. I-TASSER ^87– 89^). However these methods are generally only accessible to academics while others are required to license the technologies. This is ironic as these technologies were developed in most cases with NIH and NSF funds. An alternative commercial homology modeling approach (MODELLER) was also used and generated a NS5 homology model and the top hit was also the Japanese encephalitis virus RdRp domain (PDB ID: 4HDH) compared with PDB ID: 4K6M from SWISS-MODEL ^61^. 4HDH also includes the ATP and zinc metal where the catalytic centers are. The dengue virus 3 polymerase (PDB ID: 4HHJ) ^90^ has very high sequence homology and comes up as a potential target in MODELLER, which illustrates that all these viral RNA dependent polymerases are very similar.

While it is likely that the eventual availability of crystal structures of ZIKV proteins would improve the results of docking, the homology models described here ( Figure 1, Figure 2, Supplementary material S2) represent a starting point that can be used to help prioritize compounds for testing as described previously ^4^. Proteins with templates above 25–40% sequence identity might suggest the proteins are related while below this is a twilight zone. Homology modeling is thought to fill in the gaps between proteins with x-ray structures and those with none ^91^. Experimental testing of homology models and crystal structures indicate that a similar enrichment rate can be achieved when identifying active compounds in a set decoys ^92^. Others have also described homology models that may be an excellent alternative when crystal structures are unavailable for human GPCRs ^93, 94^, and have led to the first identification of inhibitors of the *Mycobacterium tuberculosis* Topoisomerase I after virtual screening ^95, 96^ prior to the crystal structure becoming available ^97^. Certainly there are still considerable challenges using homology models such as prediction of the correct binding pose ^98^ but there are plenty of success stories ^98– 100^. While databases of homology models exist like MODBASE ^101^ and SWISS-MODEL ^50, 51, 71– 73^ neither of these have any ZIKV protein homology models at the time of writing. There are many structural genomics initiatives and yet it would seem there are few if any continuing the work of VIZIER working on flaviviruses or emerging viruses.

Availability of structures are important as the structure of the ZIKV glycoprotein could be useful for design of antibodies selective for the virus which will be critical for the development of diagnostics, and understanding antibody binding also for the use of IV immunoglobulin in pregnancy and the organization of the epitopes on viral proteins may facilitate early work in vaccine development. There are further implications for understanding the antibody binding epitopes, which are sometimes shared between different flaviviruses. Broadly protective vaccines for flaviviruses may allow the simultaneous targeting of ZIKV and related viruses such as dengue ^102^. Understanding glycosylation is therefore important. To date Asn-154 is mentioned in Faye *et al.*, ^17^ as a glycosylation site, as are Thr-170 (mucin type *O*-linked glycosylation) and other mucin sites at Thr-245 and Thr-381. Other probably O-GlcNAC attachment sites (Ser-142, Ser-227, Thr-231, Ser-304, Thr-366, and Thr-381) were also predicted. Our analysis of *N*-glycosylation with 3 different websites suggest Asn-154 also as a likely site of *N*-glycosylation in agreement with dengue virus ^18^.

Cryo-EM has been used to show how glycoprotein E dimers arrange on the surface of virions for flaviviruses including tick-borne encephalitis virus ^103^, West Nile virus ^104^, dengue virus 1 ^105^ and dengue virus 4 ^106^ ( Supplementary material S3). The early work on the tick-borne encephalitis virus ^103^ suggested 30 dimers on the surface and also pointed to how the glycoprotein E dimers can reorganize under low pH to form a trimer. The packing of the dimers in the dengue virion is different to tick-borne encephalitis with the glycoprotein E dimers showing 30 ‘herringbone rafts’ each containing three dimers to result in 180 copies of the protein ^67, 107^. West Nile virus again has a different arrangement with 60 trimers shown in the structure of the immature virus ^104^ ( Supplementary material S4). Even between the dengue serotypes 1, 2 and 4 for which there are cryo-EM structures ^105, 106^ it is apparent while the rafts are very similar (as are the sequence identities [60%]) there is a different charge distribution of the surface of each. Dengue serotype 2 had larger continuous patches of positive charges which was proposed to enable improved binding to heparan sulfate. This might also be the case for ZIKV in that the charge pattern is again different and could be key for vaccine development. The availability of virion structures makes it feasible to understand structure function of the complete virus such as assessment of membrane curvature and how organization of membrane proteins affects this ^43^.

A model of the Zika virion was constructed as an illustration using the homology model of the glycoprotein E dimer ( Figure 3). While the combined protein sequence of glycoprotein E and the immunoglobulin like domain is closest to dengue virus 1 (57 percent identity, Table 1) the closest template was for the crystal structure of the Japanese encephalitis virus envelope protein (53.12 percent identity, Table 2). This would suggest the virion should more closely resemble that of dengue virus 1, while producing a homology model based on a more distant virus might not be ideal. The homology model of glycoprotein E developed for the mature conformation in this study is significantly different from that developed previously for the immature conformation ( Figure 4). The proposed binding site centered around residues 270-277 appears shallower in the mature conformation and this would certainly affect the kinds of molecules that it could interact with. It might also point to the need to interfere with the immature conformation as preferable versus the mature conformation. Ultimately perhaps this model of the Zika virion could help us understand how drugs could access the virus. Viruses affecting pregnancy, like say Varicella which causes microcephaly and other developmental problems ^106, 107^, are often treated with IV immunoglobulin, i.e. antibodies, as well as antivirals to reduce the effect of the virus (or to avoid infection if given soon after exposure). The models could help us design combination approaches possibly targeting multiple proteins that might prevent drug resistance from occurring also.

Does having the homology models and the virion illustration help understand function? Well, the surface charge pattern might be inferred from the homology model and could be compared with dengue and other filoviruses for which there are cryo-EM structures. This may in turn present opportunities for vaccine design by indicating accessible surfaces and properties, allowing mapping of epitopes, design of accessible fragments and peptides for vaccine/diagnostic design. Vaccines themselves might be the only way to avoid the inevitable, otherwise, simply reducing the spread of ZIKV would just delay it. Women ultimately may just want to ‘get it over with’ and have ZIKV before they get pregnant and hope there is lasting immunity.

In summary, in the absence of crystal structures for any of the proteins comprising the ZIKV, we are left to attempt to construct homology models which we have done using the freely available SWISS-MODEL server. Further preparation of these models required freely available and commercial tools. In the case of the ZIKV glycoprotein E homology model, this has the added benefit of enabling the construction of a full virion. By comparing the Zika virion to the existing structures for other flaviviruses we can see similarities and differences on the surface ( Supplementary material S4, Supplementary material S5). This relatively crude approach could help to understand how we might develop antivirals and vaccines against it. In addition we now provide homology models as a starting point for (small and large scale) docking studies and further evaluation which may complement other modeling efforts for ZIKV ^110^. Ultimately the results of their use can be compared with using ZIKV crystal structures once generated.

**Notes added while in review**

Since the initial publication of this article several cryo-EM and crystal structures have been published for ZIKV, including candidate targets for inhibitor medicinal chemistry optimization and vaccine design ^112– 115^. Detailed comparisons between our homology models and the experimental structures will be the subject of future comparisons with the homology models described herein. It should be noted that the prediction for the site of glycosylation of glycoprotein E was experimentally verified by Sirohi *et al.* ^112^, We would also point out the utility of this work through the World Community Grid we have started the OpenZika project ^116– 118^ which is using these homology models, template structures and crystal structures to dock millions of molecules (with AutoDock Vina ^119– 121^) to identify compounds for testing against Zika with collaborators. Both the docking and activity screening results ( http://openzika.ufg.br/experiments/) will be open to the community and published in due course. An obvious utility of this entire exercise is that, by testing, iterating and tweaking the methodologies each time (e.g. between Ebola and ZIKV) the open science community becomes better prepared for the next global pathogen emergency.

## Supplementary material

Supplementary material S1 (Alignments of ZIKV to other proteins), S2 (Alignment of individual ZIKV proteins for homology models), S4 (ZIKV versus dengue virion animation), and S5 (Published flavivirus cryo-EM structures [not to scale]).

Supplementary material S3. PDB files for ZIKV homology models.
