# Supplementary material for: Illustrating and homology modeling the proteins of the Zika virus
Source: F1000Res. 2016 Sep 1;5:275. Originally published 2016 Mar 3. [Version 2] doi: 10.12688/f1000research.8213.2 (PMC5040154; doi:10.12688/f1000research.8213.2)

**Supplementary material**

**Research Note**

**Illustrating and homology modeling the proteins of the Zika virus**

Sean Ekins^1,2,3^, John Liebler ^4^, Bruno J. Neves^5^, Warren Lewis^6^, Megan Coffee^7^, Rachelle Bienstock^8^, Christopher Southan^9^ and Carolina H. Andrade^5^

^1^ Collaborations in Chemistry, 5616 Hilltop Needmore Road, Fuquay-Varina, NC 27526, USA.

^2^ Collaborations Pharmaceuticals, Inc., 5616 Hilltop Needmore Road, Fuquay-Varina, NC 27526, USA.

^3^ Collaborative Drug Discovery, Inc., 1633 Bayshore Highway, Suite 342, Burlingame, CA 94010, USA

^4^ Art of the Cell, 40 Putzel Ave, Guilford, CT 06437 USA

^5^ LabMol - Laboratory for Molecular Modeling and Drug Design, Faculty of Pharmacy, Federal University of Goias, Goiânia, GO, 75605-170, Brazil

^6^ Washington University School of Medicine, Department of Medicine, St. Louis, MO 63110

^7^ The International Rescue Committee, New York, NY, USA

^8^ RJB Computational Modeling LLC, 300 Pitch Pine Lane, Chapel Hill, North Carolina, 27514-1747, USA

^9^ IUPHAR/BPS Guide to PHARMACOLOGY, Centre for Integrative Physiology, University of Edinburgh, Hugh Robson Building, Edinburgh, EH8 9XD, UK

* To whom correspondence should be addressed. Sean Ekins, E-mail address: ekinssean@yahoo.com, Phone: +1 215-687-1320, Twitter: @collabchem

Supplementary material S1. Search of ZIKV polyprotein against PDB (NCBI BLAST server, 24 Feb). The upper graphic is the conserved domain mark-up. The lower graphic shows the high-scoring matches to PDB entries, with the red lines indicating 55 to 70% sequence identity.


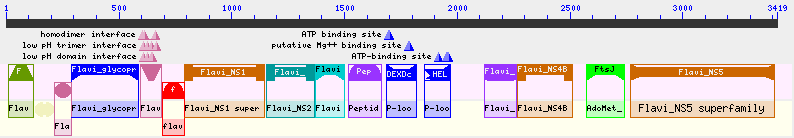

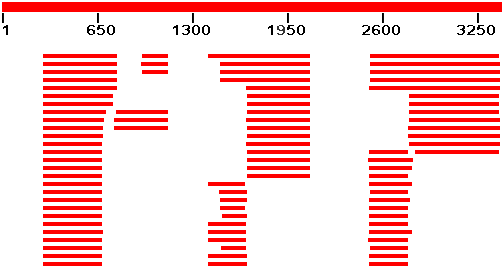


Supplementary material S2. Alignments for homology modeling

**NS5**

Target

KYEEDVNLGSGTRAVVSCAEAPNMKIIGNRIERIRSEHAETWFFDENHPYRTWAYHGSYEAPTQGSASSLINGVVRLLSK

4k6m.1.A

KYEEDVNLGSGTRAVGKGEVHSNQEKIKKRIQKLKEEFATTWHKDPEHPYRTWTYHGSYEVKATGSASSLVNGVVKLMSK

Target

PWDVVTGVTGIAMTDTTPYGQQRVFKEKVDTRVPDPQEGTRQVMSMVSSWLWKELGKHKRPRVCTKEEFINKVRSNAALG

4k6m.1.A

PWDAIANVTTMAMTDTTPFGQQRVFKEKVDTKAPEPPAGAKEVLNETTNWLWAYLSREKRPRLCTKEEFIKKVNSNAALG

Target

AIFEEEKEWKTAVEAVNDPRFWALVDKEREHHLRGECQSCVYNMMGKREKKQGEFGKAKGSRAIWYMWLGARFLEFEALG

4k6m.1.A

AVFAEQNQWSTAREAVDDPRFWEMVDEERENHLRGECHTCIYNMMGKREKKPGEFGKAKGSRAIWFMWLGARYLEFEALG

Target

FLNEDHWMGRENSGGGVEGLGLQRLGYVLEEMSRIPGGRMYADDTAGWDTRISRFDLENEALITNQMEKGHRALALAIIK

4k6m.1.A

FLNEDHWLSRENSGGGVEGSGVQKLGYILRDIAGKQGGKMYADDTAGWDTRITRTDLENEAKVLELLDGEHRMLARAIIE

Target

YTYQNKVVKVLRPAEKGKTVMDIISRQDQRGSGQVVTYALNTFTNLVVQLIRNMEAEEVLEMQDLWLLRRSEK--VTNWL

4k6m.1.A

LTYRHKVVKVMRPAAEGKTVMDVISREDQRGSGQVVTYALNTFTNIAVQLVRLMEAEGVIGPQHLEQLPRKNKIAVRTWL

Target

QSNGWDRLKRMAVSGDDCVVKPIDDRFAHALRFLNDMGKVRKDTQEWKPSTGWDNWEEVPFCSHHFNKLHLKDGRSIVVP

4k6m.1.A

FENGEERVTRMAISGDDCVVKPLDDRFATALHFLNAMSKVRKDIQEWKPSHGWHDWQQVPFCSNHFQEIVMKDGRSIVVP

Target

CRHQDELIGRARVSPGAGWSIRETACLAKSYAQMWQLLYFHRRDLRLMANAICSSVPVDWVPTGRTTWSIHGKGEWMTTE

4k6m.1.A

CRGQDELIGRARISPGAGWNVKDTACLAKAYAQMWLLLYFHRRDLRLMANAICSAVPVDWVPTGRTSWSIHSKGEWMTTE

Target

DMLVVWNRVWIEENDHMEDKTPVTKWTDIPYLGKREDLWCGSLIGHRPRTTWAENIKNTVNMVRRIIGDEEKYMDYLSTQ

4k6m.1.A

DMLQVWNRVWIEENEWMMDKTPITSWTDVPYVGKREDIWCGSLIGTRSRATWAENIYAAINQVRAVIG-KENYVDYMTSL

Target

VRY

4k6m.1.A

RRY

**FtsJ**

####

Target

VSRGSAKLRWLVERGYLQPYGKVIDLGCGRGGWSYYAATIRKVQEVKGYTKGGPGHEEPMLVQSYGWNIVRLKSGVDVFH

2oy0.1.A

VSRGTAKLRWLVERRFLEPVGKVIDLGCGRGGWCYYMATQKRVQEVRGYTKGGPGHEEPQLVQSYGWNIVTMKSGVDVFY

Target

MAAEPCDTLLCDIGESSSSPEVEEARTLRVLSMVGDWLEKRPGAFCIKVLCPYTSTMMETLERLQRRYGGGLVRVPLSRN

2oy0.1.A

RPSECCDTLLCDIGESSSSAEVEEHRTIRVLEMVEDWLHRGPREFCVKVLCPYMPKVIEKMELLQRRYGGGLVRNPLSRN

Target

STHEMYWVSGAK

2oy0.1.A

STHEMYWVSRA-

**NS4B**

Target

NELGWLERTKSDLSHLMGRREEGATIGFSMDIDLRPASAWAIYAALTTFITPAVQHAVTTSYNNYSLMAMATQAGVLFGM

3bii.1.B

--------------------------------------------------------------------------------

Target

GKGMPFYAWDFGVPLLMIGCYSQLTPLTLIVAIILLVAHYMYLIPGLQAAAARAAQKRTAAGIMKNPVVDGIVVTDIDTM

3bii.1.B

----------------------------------------------------------TFTGKVRNHN-LGDSVNALT-L

Target

T-IDPQVEKKMGQVLLIAVAVSSAILSRTAWGWGEAGALITAATSTLWEGSPNKYWNSSTATSLCNIFRGSYLAGASLIY

3bii.1.B

EHYPGMTEKALAEIVD----------------------------------------------------------------

Target

TVTRNA

3bii.1.B

------

**NS4A**

|  |  |
| --- | --- |

Target

GVMEALGTLPGHMTERFQEAIDNLAVLMRAETGSRPYKAAAAQLPETLETIMLLGLLGTVSLGIFFVLMRNKGIGKMGFG

3anw.1.B

-----LTLLPEGLYERAEFYAYYLENYVRLNPRE----------------------------------------------

Target

MVTLGASAWLMWLSEIEPARIACVLIVVFLLLVVLIPEPEKQRSPQDNQMAIIIMVAVGLLGLI

3anw.1.B

----------------------------------------------------------------

**HELICc**

Target

GKTVWFVPSVRNGNEIAACLTKAGKRVIQLSRKTFETEFQKTKHQEWDFVVTTDISEMGANFKADRVIDSRRCLKPVIL-

2bhr.1.A

GKTVWFVPSIKAGNDIAACLRKNGKKVIQLSRKTFDSEYIKTRTNDWDFVVTTDISEMGANFKAERVIDPRRCMKPVILT

Target

DG-ERVILAGPMPVTHASAAQRRGRIGRNPNKPGDEYLY

2bhr.1.A

DGEERVILAGPMPVTHSSAAQRRGRVGRNPKNENDQYIY

**DEXDc**

Target

PSMLKKKQLTVLDLHPGAGKTRRVLPEIVREAIKTRLRTVILAPTRVVAAEMEEALRGLPVRYMTTAVNVTHSGTEIVDL

2v8o.1.A

PEMLKKRQLTVLDLHPGAGKTRRILPQIIKDAIQKRLRTAVLAPTRVVAAEMAEALRGLPVRYLTPAVQREHSGNEIVDV

Target

MCHATFTSRLLQPIRVPNYNLYIMDEAHFTDPSSIAARGYISTRVEMGEAAAIFMTATPPGTRDAF

2v8o.1.A

MCHATLTHRLMSPLRVPNYNLFVMDEAHFTDPASIAARGYIATRVEAGEAAAIFMTATPPGTSDPF

**Peptidase S7**

Target

TTDGVYRVMTRRLLGSTQVGVGVMQEGVFHTMWHVTKGSALRSGEGRLDPYWGDVKQDLVSYCGPWKLDAAWDGHSEVQL

2yol.1.A

TTTGVYRIMTRGLLGSYQAGAGVMVEGVFHTLWHTTKGAALMSGEGRLDPYWGSVKEDRLCYGGPWKLQHKWNGHDEVQM

Target

LAVPPGERARNIQTLPGIFKTKDGDIGAVALDYPAGTSGSPILDKCGRVIGLYGNGVVIKNGSYVSAITQG

2yol.1.A

IVVEPGKNVKNVQTKPGVFKTPEGEIGAVTLDYPTGTSGSPIVDKNGDVIGLYGNGVIMPNGSYISAIVQG

**NS2B**

Target

PSEVLTAVGLICALAGGFAKADIEMAGPMAAVGLLIVSYVVSGKSVDMYIERAGDITWEKDAEVTGNSPRLDVALDESGD

2fp7.1.A

---------------------------------------------TDMWIERTADITWESDAEITGSSERVDVRLDDDGN

Target

FSLVEDDGPPMREIILKVVLMTICGMNPIAIPFAAGAWYVYVKTGKR

2fp7.1.A

FQLMNDPGAPWK-----------------------------------

**NS2A**

Target

GVLVILLMVQEGLKKRMTTKIIISTSMAVLVAMILGGFSMSDLAKLAILMGATFAEMNTGGDVAHLALIAAFKVRPALLV

4v8p.1.g

----------------------------------FGITCVEDLIHEITTVGPHFKEANNF--------LWPFKLDT----

Target

SFIFRANWTPRESMLLALASCLLQTAISALEGDLMVLINGFALAWLAIRAMVVPRTDNITLAILAALTPLARGTLLVAWR

4v8p.1.g

--------------------------------------------------------------------------------

Target

AGLATCGGFMLLSLKGKGSVKKNLPFVMALGLTAVRLVDPINVVGLLLLTRSGKR

4v8p.1.g

-------------------------------------------------------

**NS1**

Target

VGCSVDFSKKETRCGTGVFVYNDVEAWRDRYKYHPDSPRRLAAAVKQAWEDGICGISSVSRMENIMWRSVEGELNAILEE

4o6d.1.A

TGCAIDISRQELRCGSGVFIHNDVEAWMDRYKYYPETPQGLAKIIQKAHKEGVCGLRSVSRLEHQMWEAVKDELNTLLKE

Target

NGVQLTVVVGSVKNPMWRGPQRLPVPVNELPHGWKAWGKSYFVRAAKTNNSFVVDGDTLKECPLKHRAWNSFLVEDHGFG

4o6d.1.A

NGVDLSVVVEKQEGMYKSAPKRLTATTEKLEIGWKAWGKSILFAPELANNTFVVDGPETKECPTQNRAWNSLEVEDFGFG

Target

VFHTSVWLKVREDYSLECDPAVIGTAVKGKEAVHSDLGYWIESEKNDTWRLKRAHLIEMKTCEWPKSHTLWTDGIEESDL

4o6d.1.A

LTSTRMFLKVRESNTTECDSKIIGTAVKNNLAIHSDLSYWIESRLNDTWKLERAVLGEVKSCTWPETHTLWGDGILESDL

Target

IIPKSLAGPLSHHNTREGYRTQMKGPWHSEELEIRFEECPGTKVHVEETCGTRGPSLRSTTASGRVIEEWCCRECTMPPL

4o6d.1.A

IIPVTLAGPRSNHNRRPGYKTQNQGPWDEGRVEIDFDYCPGTTVTLSESCGHRGPATRTTTESGKLITDWCCRSCTLPPL

Target

SFRAKDGCWYGMEIRPRKEPESNLVRSMVTAGS

4o6d.1.A

RYQTDSGCWYGMEIRPQRHDEKTLVQSQVNA--

**E Stem**

Target

GKAFEATVRGAKRMAVLGDTAWDFGSVGGALNSLGKGIHQIFGAAFKSLFGGMSWFSQILIGTLLMWLGLNTKNGSISLM

3j2p.1.A GQMIETTMRGAKRMAILGDTAWDFGSLGGVFTSIGKALHQVFGAIYGAAFSGVSWIMKILIGVIITWIGMNSRSTSLSVS

Target

CLALGGVLIFLSTAVSA

3j2p.1.A

LVLVGVVTLYLGVMVQA

**Glycoprotein M**

Target

AVTLPSHSTRKLQTRSQTWLESREYTKHLIRVENWIFRNPGFALAAAAIAWLLGSSTSQKVIYLVMILLIAPAYS

3j27.1.F

SVALVPHVGMGLETATETWMSSEGAWKHAQRIETWILRHPGFTIMAAILAYTIGTTHFQRALIFILLTAVAPSMT

**Propep**

Target

TRRGSAYYMYLDRNDAGEAISFPTTLGMNKCYIQIMDLGHMCDATMSYECPMLDEGVEPDDVDCWCNTTSTWVVYGTCHH

3c5x.1.B

TTRNGEPHMIVSRQEKGKSLLFKTEDGVNMCTLMAMDLGELCEDTITYKCPLLRQN-EPEDIDCWCNSTSTWVTYGTC--

Target

KKGEARRSR

3c5x.1.B

---------

**Capsid**

Target

KKSGGFRIVNMLKRGVARVSPFGGLKRLPAGLLLGHGPIRMVLAILAFLRFTAIKPSLGLINRWGSVGKKEAMEIIKKFK

1sfk.1.C

-----------------RVLSLTGLKRAMLSLIDGRGPTRFVLALLAFFRFTAIAPTRAVLDRWRSVNKQTAMKHLLSFK

Target

KDLAAMLRIINARKEKKRRGADTSVGIVGLLLTTAMA

1sfk.1.C

KELGTLTSAINRR------------------------

**Glycoprotein E**

Target

IRCIGVSNRDFVEGMSGGTWVDVVLEHGGCVTVMAQDKPTVDIELVTTTVSNMAEVRSYCYEASISDMASDSRCPTQGEA

3p54.1.A

--CLGMGNRDFIEGASGATWVDLVLEGDSCLTIMANDKPTLDVRMINIEASQLAEVRSYCYHASVTDISTVARCPTTGEA

Target

YLDKQSDTQYVCKRTLVDRGWGNGCGLFGKGSLVTCAKFACSKKMTGKSIQPENLEYRIMLSVHGSQHSGMIVNDTGHET

3p54.1.A

HNEKRADSSYVCKQGFTDRGWGNGCGFFGKGSIDTCAKFSCTSKAIGRTIQPENIKYKVGIFVHGTTTSENHGNYSAQVG

Target

DENRAKVEITPNSPRAEATLGGFGSLGLDCEPRTGLDFSDLYYLTMNNKHWLVHKEWFHDIPLPWHAGADTGTPHWNNKE

3p54.1.A

ASQAAKFTVTPNAPSVTLKLGDYGEVTLDCEPRSGLNTEAFYVMTVGSKSFLVHREWFHDLALPWTSPSSTA---WRNRE

Target

ALVEFKDAHAKRQTVVVLGSQEGAVHTALAGALEAEMDGAKGRLSSGHLKCRLKMDKLRLKGVSYSLCTAAFTFTKIPAE

3p54.1.A

LLMEFEGAHATKQSVVALGSQEGGLHQALAGAIVVEYSSSV-MLTSGHLKCRLKMDKLALKGTTYGMCTEKFSFAKNPVD

Target

TLHGTVTVEVQYAGTDGPCKVPAQMAVDMQTLTPVGRLITANPVITESTENSKMMLELDPPFGDSYIVIGVGEKKITHHW

3p54.1.A

TGHGTVVIELSYSGSDGPCKIPIVSVASLNDMTPVGRLVTVNPFVATSSANSKVLVEMEPPFGDSYIVVGRGDKQINHHW

Target

HRSGSTI

3p54.1.A

HKAGSTL

Supplementary material S4. ZIKV versus dengue virion animation.


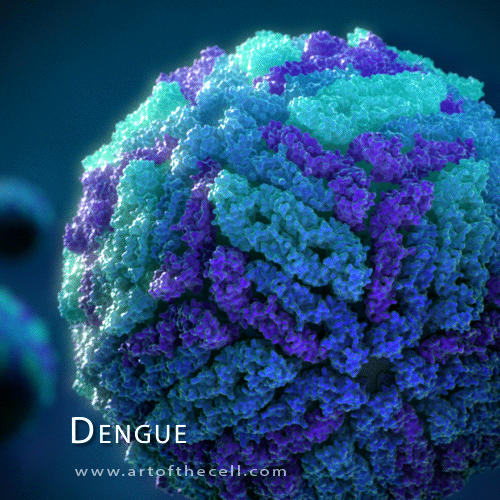


Supplementary material S5. Published flavivirus cryo-EM structures (not to scale). A Immature dengue 1 [103], B Mature dengue 1 [105], C Mature dengue 4 [106], D Immature West Nile virus [104]. Images from EMDataBank [111].

A
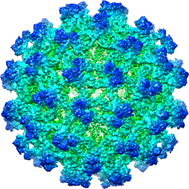
 B
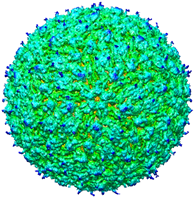


C
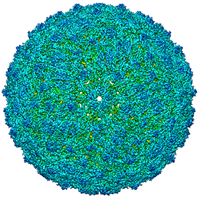
 D
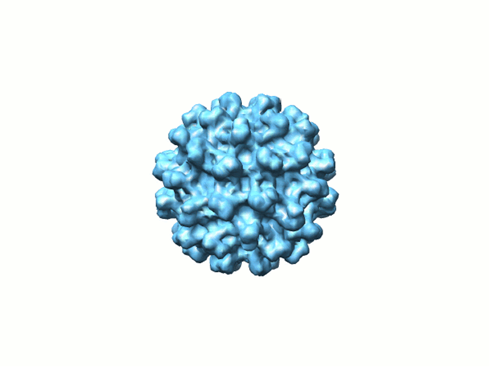

Supplement: Supplementary file 1 [file f1000research-5-10258-s0000.tgz › 14167f91-588a-42f7-b987-36baf95d8f88.docx]
