# Supplementary material for: Illustrating and homology modeling the proteins of the Zika virus
Source: F1000Res. 2016 Sep 1;5:275. Originally published 2016 Mar 3. [Version 2] doi: 10.12688/f1000research.8213.2 (PMC5040154; doi:10.12688/f1000research.8213.2)
Supplement: Peer review history

# Peer review: Referee response for version 2

**DOI:** 10.5256/f1000research.10258.r16629

**Published:** 2016-09-27

**Authors:** Bruno Botta

**Affiliations:** 1 Department of Chemistry and Pharmaceutical Technology, Sapienza University of Rome, Rome, Italy

The revised manuscript can be indexed. It has been improved accordingly.

I have read this submission. I believe that I have an appropriate level of expertise to confirm that it is of an acceptable scientific standard.

# Peer review: Referee response for version 2

**DOI:** 10.5256/f1000research.10258.r16006

**Published:** 2016-09-16

**Authors:** Andras Fiser

**Affiliations:** 1 Albert Einstein College of Medicine, Bronx, NY, USA

The Authors clarified many of the points I raised and I approve the manuscript.

I have read this submission. I believe that I have an appropriate level of expertise to confirm that it is of an acceptable scientific standard.

# Peer review: Referee response for version 1

**DOI:** 10.5256/f1000research.8833.r15364

**Published:** 2016-08-02

**Authors:** Bruno Botta

**Affiliations:** 1 Department of Chemistry and Pharmaceutical Technology, Sapienza University of Rome, Rome, Italy

The work of Sean Ekins and colleagues describes an attempt to predict the 3D structure of a number of Zika Virus proteins by homology modeling.

 Infections by Zika virus are seriously preoccupying the population and governments of endemic countries, with particular attention to pregnant women. Indeed, the most serious complications of infections by Zika virus have been observed in pregnant women (i.e. microencephalic fetus). However, the evidence that pregnant women are most affected by Zika virus infection, set some difficulties in developing focused therapeutic strategies, as nicely discussed by the authors. However, in this respect, which is the scenario proposed by authors for a candidate inhibitor of Zika virus replication. Why this molecule should be useful, and how pregnant women could deal with the administration of the drug?

 Overall, the work is well written and organized in a clear and rational way, even if no experimental validation of the work carried out *in silico* is proposed. Accordingly, conclusions of this work are merely speculative. My major concern is on the usability of the protein structures for drug design or virtual screening, as claimed by the authors in the abstract and discussion. Indeed, sequence identity between template and target sequences is rather low in some cases (< 60 %). Moreover, the atomistic detail of the active site and its conformation may vary noticeably in the models depending on the program used, the force field, the level of refinement (algorithm, steps, solvent model, …) and the quality of the template structure. All these variables should be taken into consideration by the authors, and the refinement of 3D models should be at least attempted or discussed in deeper details. In my personal opinion, details on the conservation rate, sequence identity and structural similarity of the binding sites would be more helpful for drug design purposes.

 Finally, an experimental validation of at least one of the structures modeled by authors (i.e. the most promising target for drug designing studies) should be provided. In fact, analysis with PROCHECK or the use of numerical scores is not sufficient to validate a 3D structure generated by homology modeling. There is a high risk that molecular docking towards a low-resolution structure could provide unrealistic results. The paper can be accepted after revision.

I have read this submission. I believe that I have an appropriate level of expertise to confirm that it is of an acceptable scientific standard, however I have significant reservations, as outlined above.

# Response

**Published:** 2016-08-25

**Authors:** Sean Ekins

**Affiliations:** Collaborations in Chemistry, USA

**Response: Dear Prof Botta, thank you for taking the time to review and constructively comment on this manuscript.**

 Infections by Zika virus are seriously preoccupying the population and governments of endemic countries, with particular attention to pregnant women. Indeed, the most serious complications of infections by Zika virus have been observed in pregnant women (i.e. microencephalic fetus). However, the evidence that pregnant women are most affected by Zika virus infection, set some difficulties in developing focused therapeutic strategies, as nicely discussed by the authors. However, in this respect, which is the scenario proposed by authors for a candidate inhibitor of Zika virus replication. Why this molecule should be useful, and how pregnant women could deal with the administration of the drug?

**Response – If we understand the question correctly - any drug for Zika should be safe for all including pregnant women. If they have a Zika infection it would be important to eradicate the virus as soon as possible so that the fetus perhaps is not exposed to the virus.**

 Overall, the work is well written and organized in a clear and rational way, even if no experimental validation of the work carried out *in silico* is proposed.

**Response: This work was initiated in Feb 2016, months before the first crystal structures and experimental in vitro systems were published. Our goal was to build protein models that we would eventually use and which others could use in parallel, to start docking-based virtual screening and identify VS and experimental hits, to initiate the drug discovery cascade.**

 Accordingly, conclusions of this work are merely speculative.

**Response: A modeling study, by definition is speculative and does not claim otherwise.  However, in good faith, we provided models which did not exist. The fact that they have since gone on to form the basis of the OpenZika Project (manuscript submitted, http://openzika.ufg.br) and have been used to suggest compounds which are now being tested argues for their utility. Notwithstanding, we acknowledge that Zika protease, helicase and glycoprotein E experimental structures have now been elucidated and deposited in PDB.**

 My major concern is on the usability of the protein structures for drug design or virtual screening, as claimed by the authors in the abstract and discussion. Indeed, sequence identity between template and target sequences is rather low in some cases (< 60 %).

**Response – As expected for the enzyme functions, the local active site identity and similarity scores are higher than indicated by the full-length identity %. Thus, a reasonable pocket model for docking is the primary goal, not the structural accuracy for the whole protein.  We would also add that any team using our models would doubtless take a pragmatic approach that would include running various types of internal controls for virtual screening studies and confirmatory screening experiments (e.g. running the Dengue virus templates alone and checking for enrichment of established Dengue actives against the purified proteins). They would also probably prioritize their effort on the models with highest template similarity. Note also that, the first reviewer Dr. Fiser accepts that our similarity values are high enough for reliable modelling.**

 Moreover, the atomistic detail of the active site and its conformation may vary noticeably in the models depending on the program used, the force field, the level of refinement (algorithm, steps, solvent model, …) and the quality of the template structure.

**Response – We agree that this is one of the challenges with this approach, but there were no other options without a single crystal structure for Zika protein when we started this work and submitted it.**

 All these variables should be taken into consideration by the authors, and the refinement of 3D models should be at least attempted or discussed in deeper details. In my personal opinion, details on the conservation rate, sequence identity and structural similarity of the binding sites would be more helpful for drug design purposes.

**Response: We agree with your point. We are now refining the 3D models using molecular dynamics simulations. However, this will be the subject of work on going, taking an in depth approach to each structure and comparison to crystal structures when available. Our main goal in this paper which we submitted in Feb 2016 (and made available early March) was to provide the scientific community with the first 3D models of Zika proteins.**

 Finally, an experimental validation of at least one of the structures modeled by authors (i.e. the most promising target for drug designing studies) should be provided. In fact, analysis with PROCHECK or the use of numerical scores is not sufficient to validate a 3D structure generated by homology modeling.

**Response – We agree that the experimental validation of the protein structures is of upmost importance in drug discovery programs, but this was not the scope of this paper. Again, our main goal was to generate homology models for the key proteins, which none crystal was available at the moment of the submission, and these models could be taken to initiate ZIKV antiviral drug discovery using both high throughput screens as well as structure-based design. Moreover, these structures were the core to initiate the World Community Grid project called OpenZika ( https://www.worldcommunitygrid.org/research/zika/overview.do), which is a global research project to accelerate the discovery of an antiviral against the Zika virus.**

**PROCHECK was originally developed to evaluate the stereochemical quality of experimentally determined protein structures (Laskowski R A, MacArthur M W, Moss D S, Thornton J M (1993). J. App. Cryst., 26, 283-291; Morris A L, MacArthur M W, Hutchinson E G & Thornton J M (1992). Proteins, 12, 345-364). However, PROCHECK has been widely used and reported in the literature to check the stereochemical quality of homology models, to provide numerical scores that can evaluate the statistical quality of the generated 3D protein models (See bellow the list of summarized most recent literature).  It is a quality assessment criterion and is not related to experimental validation.**

**Papers reporting the use of PROCHECK for assessing the stereochemical quality of homology models:**

**J Biomol Struct Dyn. 2016 Apr 4:1-19 [DOI: 10.1080/07391102.2015.1117397].**

**Curr Cancer Drug Targets. 2015;15(9):822-35 [PMID: 26567883].**

**Interdiscip Sci. 2015 Aug 15. In Press [DOI: 10.1007/s12539-015-0121-z].**

**Onco Targets Ther. 2015 Jul 30;8:1923-30 [DOI: 10.2147/OTT.S84200].**

**Interdiscip Sci. 2016 Mar;8(1):41-52 [DOI: 10.1007/s12539-015-0269-6].**

**J Mol Model. 2015 Mar;21(3):37. [DOI: 10.1007/s00894-015-2586-4].**

 There is a high risk that molecular docking towards a low-resolution structure could provide unrealistic results.

**Response – Yes we absolutely agree, but when we started this we had no crystal structures we had to start somewhere as a means to start to filter compounds rather than random HTS.**

**Our work has also correctly predicted the glycosylation site for glycoprotein E one of the first structures crystallized. Moreover, we have compared our models with the crystallographic structures, when they were made available. The RMSD values ranged from 0.72 to 1.8 Å, for the structures of NS1, NS3, glycoprotein E and NS2/NS3 proteins. We have also optimized the 3D models and the MolProbit scores ranged from 1.28 to 2.81 Å. MolProbit is a score that combines the clashscore, rotamer, and Ramachandran evaluations into a single score, normalized to be on the same scale as X-ray resolution. Therefore, we are optimistic that our models are reliable to start a docking-based virtual screening program in the search of a new antiviral drug, which we already initiated.**

 The paper can be accepted after revision.

**Response – Thank you!**

# Peer review: Referee response for version 1

**DOI:** 10.5256/f1000research.8833.r13009

**Published:** 2016-04-11

**Authors:** Andras Fiser

**Affiliations:** 1 Albert Einstein College of Medicine, Bronx, NY, USA

The paper describes homology models of 15 proteins encoded in the genome of Zika virus that are built by the SWISSmodel web server. A glycolysation site was also identified. Using the models of Zika glycoprotein E a complete structural model of the virion was constructed.

The practical results of the work are the 11 high quality homology models that could be used in the future for structure based drug development. I believe an interested researcher will initiate time consuming follow up studies with these models only if there is a substantial added value.

These models passed quality assessment criteria according to the authors. However a number of successful models (passing similarly well the same quality requirements) can be generated that will differ in small but essential details by e.g. side chain placement, or loop conformations. However, these differences can have a dramatic effect on the outcome of subsequent drug docking trials. These alternative models can be obtained when different softwares are used, as the authors allude to it, e.g. using Modeller vs SWISSModel, as these use different forcefields and restraints to generate models. Therefore, the authors should consider providing a more insightful result by running several different modeling programs or using alternative templates (see 4., below), and comparing the results, identifying similarly modeled parts of these models and providing a set of possible solutions for subsequent studies.  

A battery of model quality checks were performed and additional energy minimization. However the overall high sequence identities between the target proteins and their respective templates (55% and up,) ensure that these models are highly reliable.  

1. Therefore the extensive reporting on Ramachandran plots (Figure 3) is not adding much to the results, it can be transferred to supplementary material.
2. Similarly, Table 2 can be shrunk by eliminating many details of PROCHECK results.
3. However the information on templates should be reported in the table, instead listing them extensively in the text.
4. Template selection was purely based on sequence similarity requirements. Given how influential this step and how few cases require attention in this specific study, one could imagine to perform a more detailed quality check of potential templates. For instance, if several templates are available in the same sequence identity range, one should consider picking the one wit the best resolution etc. Figure 4 exposes this dilemma where two isoforms of the same template can results models with very significant differences.
5. Energy refinement rarely, if ever, improves model quality and its use here should be better justified.
6. Some statements require attention e.g in Abstract: “Eleven out of 15 models pass our criteria for selection”. What selection? This must be referring to quality or accuracy of models, and should be rephrased accordingly.

I have read this submission. I believe that I have an appropriate level of expertise to confirm that it is of an acceptable scientific standard, however I have significant reservations, as outlined above.

# Response

**Published:** 2016-08-25

**Authors:** Sean Ekins

**Affiliations:** Collaborations in Chemistry, USA

**Dear Prof Fiser, thank you for taking the time to review and constructively comment on this manuscript.**

 The paper describes homology models of 15 proteins encoded in the genome of Zika virus that are built by the SWISSmodel web server. A glycolysation site was also identified. Using the models of Zika glycoprotein E a complete structural model of the virion was constructed.

 The practical results of the work are the 11 high quality homology models that could be used in the future for structure based drug development. I believe an interested researcher will initiate time consuming follow up studies with these models only if there is a substantial added value.

**Response: Thank you!** **We would also point out the utility of this work is not only reflected in the high level of accesses, downloads and citations, but also for initiating the World Community Grid as the OpenZika project, which was launched in May, 2016 (https://www.worldcommunitygrid.org/research/zika/overview.do)**

 These models passed quality assessment criteria according to the authors. However a number of successful models (passing similarly well the same quality requirements) can be generated that will differ in small but essential details by e.g. side chain placement, or loop conformations. However, these differences can have a dramatic effect on the outcome of subsequent drug docking trials. These alternative models can be obtained when different softwares are used, as the authors allude to it, e.g. using Modeller vs SWISSModel, as these use different force fields and restraints to generate models. Therefore, the authors should consider providing a more insightful result by running several different modeling programs or using alternative templates (see 4., below), and comparing the results, identifying similarly modeled parts of these models and providing a set of possible solutions for subsequent studies.  

**Response: We would agree that certainly an exhaustive analysis of different methods was not undertaken. That was not our goal. We attempted to build models that we could then put into practice and provide to the community for them to use knowing the possible caveats.**

 A battery of model quality checks were performed and additional energy minimization. However the overall high sequence identities between the target proteins and their respective templates (55% and up,) ensure that these models are highly reliable.  

**Response: Thank you!**

1. Therefore the extensive reporting on Ramachandran plots (Figure 3) is not adding much to the results, it can be transferred to supplementary material.
**Response: Actually many publications use these plots and they may provide some insights on model quality so we have opted to retain them in the body of the manuscript, as the F1000Research journal does not have a limit for figures.**
2. Similarly, Table 2 can be shrunk by eliminating many details of PROCHECK results.
**Response: This is an internet publication we are not constrained by pages or page charges and these numbers are informative to readers. **
3. However the information on templates should be reported in the table, instead listing them extensively in the text.
**Response: We agree and we believe that it is neater and more accessible in a table. Therefore, we have added the information on templates in Table 2 and removed this information from the text.**
** **
4. Template selection was purely based on sequence similarity requirements. Given how influential this step and how few cases require attention in this specific study, one could imagine to perform a more detailed quality check of potential templates. For instance, if several templates are available in the same sequence identity range, one should consider picking the one with the best resolution etc. Figure 4 exposes this dilemma where two isoforms of the same template can results models with very significant differences.
**Response: We totally agree – some decisions had to be made as a trade-off to get the models built in a reasonable time to put into use. We could have spent months building models by which time the x-ray structures would have been out. We had models of the proteins and the virion at least 1 month before the first cryo-EM structure. One month is a long time in drug discovery, in which we could be finding molecules for testing.**
5. Energy refinement rarely, if ever, improves model quality and its use here should be better justified.
**Response: We totally agree with you, Dr. Fiser. Energy minimization does not improve model quality. The word “refined”in the paper was a mistake and we have corrected it now. We are using some servers, such as KoBaMIN server, to perform structure refinement and to improve models quality, but this will be subject of another manuscript. Our main goal in this first paper was to provide the scientific community with homology models for the key proteins, which none crystal was available at the moment of the submission of this paper, and these models could be taken to initiate ZIKV antiviral drug discovery using both high throughput screens as well as structure-based design. Moreover, these structures were the core to initiate the World Community Grid project called OpenZika ( https://www.worldcommunitygrid.org/research/zika/overview.do), which is a global research project to accelerate the discovery of an antiviral against the Zika virus**.
6. Some statements require attention e.g in Abstract: “Eleven out of 15 models pass our criteria for selection”. What selection? This must be referring to quality or accuracy of models, and should be rephrased accordingly.
**Response: OK Thank you!** **Changed to -** **Eleven out of 15 models pass our model quality criteria for their further use.**
